# *In vitro* evidence that plasma of women with eclampsia disrupts the blood-brain barrier

**DOI:** 10.3389/fphys.2026.1778955

**Published:** 2026-04-27

**Authors:** Jesenia Acurio, Felipe Troncoso, Esthefanny Escudero-Guevara, Hermes Sandoval, Belen Ibañez, Manu Vatish, Pablo Torres-Vergara, Lina Bergman, Carlos Escudero

**Affiliations:** 1 Vascular Physiology Laboratory, Department of Basic Sciences, Universidad del Bío-Bío, Chillán, Chile; 2 Doctorade Program in Biomedical Sciences, Universidad de Talca, Talca, Chile; 3 Doctorade Program in Veterinarian Sciences, Universidad de Concepcion, Chillan, Chile; 4 Nuffield Department of Women’s & Reproductive Health, Women’s Centre, John Radcliffe Hospital, University of Oxford, Oxford, United Kingdom; 5 Group of Research and Innovation in Vascular Health (GRIVAS Health), Chillan, Chile; 6 Departamento de Farmacia, Facultad de Farmacia, Universidad de Concepción, Concepción, Chile; 7 Department of Obstetrics and Gynecology, Institute of Clinical Sciences, University of Gothenburg, Gothenburgh, Sweden; 8 Department of Obstetrics and Gynecology, Stellenbosch University, Cape Town, South Africa; 9 Department of Women’s and Children’s Health, Uppsala University, Uppsala, Sweden; 10 Department of Obstetrics and Gynecology, Sahlgrenska University Hospital, Gothenburg, Region Västra Götaland, Sweden; 11 Wallenberg Center of Molecular and Translational Medicine, University of Gothenburg, Gothenburg, Sweden; 12 Consortium for Research and Innovation Neurovascular (NEUROVAS), Chillan, Chile

**Keywords:** Blood-brain barrier, brain endothelial cells, eclampsia, magnesium sulfate, small extracellular vesicles

## Abstract

**Background:**

Eclampsia is a severe complication of preeclampsia involving blood-brain barrier (BBB) disruption. While small extracellular vesicles (sEVs) contribute to endothelial dysfunction in preeclampsia, their role in eclampsia remains unclear. We examined the effects of plasma and plasma-derived sEVs from women with eclampsia on BBB integrity.

**Methods:**

Plasma and plasma-sEVs were isolated from women with normotensive pregnancies (n=18), preeclampsia (n=19), preeclampsia with organ complications (n=17), and eclampsia (n=20). An *in vitro* BBB model based on the culture of human brain endothelial cells was used to evaluate electrical resistance (TEER) and Dextran 70 kDa permeability in the presence of women’s plasmas or plasma-sEVs. sEVs cargo of relevant proteins involved in BBB regulation, eNOS, and TNF-α, were analyzed.

**Results:**

Plasma from women with eclampsia disrupted the BBB, with marked reductions in TEER and increased permeability compared to normotensive controls, preeclampsia, and preeclampsia with organ complications. Moreover, plasma-sEVs of women with eclampsia caused a drop in TEERs but less BBB permeability than plasma-sEVs from normotensive controls or preeclampsia. Lower levels of eNOS and TNF-α in eclampsia-derived sEVs compared to normotensive controls were found.

**Conclusions:**

We report the critical role of circulating plasma factors in the disruption of the BBB during eclampsia. Although plasma-derived sEVs induce some alterations in barrier properties, our findings suggest they are not the main drivers of the BBB impairment observed in eclampsia, likely due to altered cargo composition.

## Introduction

Preeclampsia is a multisystem hypertensive disorder of pregnancy characterized by new-onset hypertension and multiorgan involvement after 20 weeks of gestation (Acog, 2019). Among its clinical manifestations, eclampsia is a severe complication defined by the onset of generalized tonic-clonic seizures during pregnancy. Eclampsia affects approximately 1 in 100 women with preeclampsia, with a substantially higher prevalence in low-resource settings, reaching 50 to 151 cases per 10,000 deliveries in regions such as Latin America and sub-Saharan Africa (Duley, 2009; Fishel Bartal and Sibai, 2022). Furthermore, beyond its acute life-threatening effects, eclampsia has been linked to various neurological complications that may persist after pregnancy, highlighting the need for early identification of at-risk women to improve immediate and future cerebrovascular outcomes (Bellamy et al., 2007; Junewar et al., 2014; Nerenberg et al., 2017; Verma et al., 2017; Basit et al., 2018; Dayan et al., 2018; Elharram et al., 2018; Adank et al., 2020; Friis et al., 2025).

Although eclampsia is clinically significant, its pathophysiology remains incompletely understood. Dysfunction of the blood-brain barrier (BBB), a highly selective, multicellular interface critical for maintaining brain homeostasis, is increasingly recognized as a key mechanism underlying eclamptic seizures and associated brain injury (Cipolla et al., 2011; Torres-Vergara et al., 2018; Bergman et al., 2021c). Initial preclinical studies demonstrated that plasma from women with preeclampsia induces BBB disruption *in vitro* (Amburgey et al., 2010; Bergman et al., 2021a). Following this, several circulating factors have been investigated as potential mediators of BBB dysfunction in preeclampsia, including tumor necrosis factor-alpha (TNF-α) (Cipolla et al., 2012; Warrington et al., 2015), activation of vascular endothelial growth factor receptor 2 (VEGFR2) (Amburgey et al., 2010; Warrington et al., 2015; Bergman et al., 2021a), angiotensin II type 1 receptor agonistic autoantibodies (AT1-AA) (Duncan et al., 2020), and small extracellular vesicles (sEVs) (Leon et al., 2021; Sandoval et al., 2024). These findings support the concept that BBB dysfunction in preeclampsia arises from one or more circulating factors, yet the precise identity of these factors remains unresolved. Importantly, the role and mechanisms of these circulating factors in eclampsia remain poorly defined.

Small extracellular vesicles (sEVs) are lipid bilayer-enclosed nanoparticles released by cells that carry bioactive cargo, including proteins, lipids, and nucleic acids, reflective of their cell of origin. Circulating levels of sEVs, theoretically released from the placenta, are elevated in preeclampsia compared to normal pregnancy (Redman and Sargent, 2008; Salomon et al., 2017; Han et al., 2020; Li et al., 2020; Leon et al., 2021; Cooke et al., 2024). Studies, including those from our group, have shown that sEVs isolated from the plasma of women with preeclampsia can impair the BBB *in vitro* (Leon et al., 2021; Sandoval et al., 2024). However, the specific cargo and mechanisms driving these effects remain largely unknown. Although thousands of sEVs candidates are potentially harmful to endothelial cells, research has shown that reduced activity of endothelial nitric oxide synthase (eNOS) in sEVs from the placentas of women with preeclampsia (Motta-Mejia et al., 2017) may contribute to the systemic endothelial dysfunction observed in preeclampsia. Currently, it is unclear whether eNOS cargo within sEVs in preeclampsia or eclampsia may impair the BBB.

Therefore, this study aimed to investigate whether plasma from women with eclampsia disrupts the BBB. We also aimed to investigate whether sEVs from the plasma of women with eclampsia can participate in this BBB alteration. Additionally, we assessed the presence of eNOS and TNF-α in sEVs cargo from women with eclampsia to explore possible mechanistic pathways. The selection of eNOS and TNF-α was based on their established roles in endothelial homeostasis and inflammatory signaling relevant to BBB integrity (Cipolla et al., 2012; Warrington et al., 2015; An et al., 2021).

## Methods

### Population

Women participating in the PROVE biobank at Tygerberg Hospital, South Africa, were eligible for inclusion in the study (Bergman et al., 2021b). Participants were included between 12/04/2018 and 31/01/2020. All variables were prospectively entered by research midwives, obtained from interviews or medical charts, and double-checked for accuracy. All women were managed according to clinical routine, including administration of MgSO_4_ in case of threatening preterm birth <32 weeks and for seizure prophylaxis in preeclampsia.

Exposures were preeclampsia, preeclampsia with organ complications without cerebral features, and eclampsia. Diagnosis of preeclampsia and eclampsia was defined using the International Society for the Study of Hypertension in Pregnancy (ISSHP) classification (Brown et al., 2018). In addition, significant proteinuria was required for the diagnosis of preeclampsia (2+ protein on a dipstick and/or urine protein/creatinine ratio above 30 mg/mmol). Preeclampsia with organ complications was defined according to a core outcome set of preeclampsia-related complications (Duffy et al., 2020).

Normotensive controls were defined as women with a pregnancy where blood pressure did not exceed 140/90 mm Hg. Exclusion criteria for the exposure and control groups were pre-existing neurological disorders and cardiovascular disease.

The research was conducted in accordance with the principles expressed in the Declaration of Helsinki and with the authorization of the respective Ethical Review Boards. Ethical approval for the inclusion of women for this study was obtained from the Stellenbosch University Health Research Ethics Committee (protocol number N18/03/034; Federal Wide Assurance number 00001372; institutional review board number: IRB0005239). All participants gave their informed consent before sample collection.

### Outcomes

#### TEER and permeability

To analyze the function of the BBB *in vitro*, we used a previously validated Transwell^®^ system with the hCMEC/D3 cell line (Weksler et al., 2013; Bergman et al., 2021a; Friis et al., 2022). This is a human brain microvascular endothelial cell line derived from healthy brain tissue of an adult epileptic woman (Weksler et al., 2013). Briefly, cell monolayers (100% confluent) were exposed to plasma (1% v/v, 12 h) or sEVs (100 μg sEVs per Transwell, 12 h) belonging to the respective experimental groups (see below). No time-course experiments were performed. Measurements of TEER (EVOM2, World Precision Instruments, USA) and cell monolayer permeability to high molecular weight fluorescent dye (Dextran 70 kDa fluorescein-5-isothiocyanate-FITC) were performed as described previously (Bergman et al., 2021a; Friis et al., 2022). Briefly, TEER was recorded as raw resistance values (Ω) subtracting cell-free insert resistance and normalized to membrane surface area (0.33 cm² for 24-well Transwell inserts) to obtain values expressed as Ω·cm². hCMEC/D3 cells were maintained on inserts for 5–7 days to allow monolayer maturation, reaching maximal raw resistance values of approximately 200 Ω (≈66 Ω·cm²). Only inserts demonstrating baseline raw resistance ≥180 Ω (≈59 Ω·cm²) were included in the study. For functional analyses, TEER responses were expressed as percentage change relative to the baseline value of each insert prior to treatment.

The hCMEC/D3 cell line is one of the most widely validated human brain endothelial cell lines for *in vitro* BBB studies (Eigenmann et al., 2013; Rahman et al., 2016). Although monoculture systems do not recapitulate the complete neurovascular unit, comparative studies indicate that co-culture with astrocytes or conditioned media does not consistently enhance barrier phenotype in this model. Therefore, hCMEC/D3 cells provide a robust and reproducible system for assessing endothelial-specific responses, while acknowledging the inherent limitations of monoculture approaches.

### Biological samples

EDTA plasma from women with normal pregnancy (n=18), preeclampsia (n=19), preeclampsia with systemic (non-cerebral) complications (n=17), and eclampsia (n=20) were obtained from the Preeclampsia Obstetric Adverse Events Biobank at the Tygerberg Obstetric Critical Care Unit in South Africa (Bergman et al., 2021b) and used for all *in vitro* experiments. For *in vitro* experiments, 1% plasma was diluted into culture medium lacking fetal calf serum (v/v), and cells were exposed to each condition for varying treatment times, depending on the analysis. For each experimental analysis, we used at least five randomly selected samples to optimize the limited amount of valuable samples across all experimental approaches.

In the case of sEVs, cell monolayers (100% confluent) were exposed to sEVs (100 μg sEVs per Transwell, 12 h) belonging to the respective experimental groups. The applied doses corresponded approximately to 2.4 × 10^7^, 3.0 × 10^7^, 4.5 × 10^7^, and 3.4 × 10^7^ particles/cm² for eclampsia, preeclampsia with complications, preeclampsia without complications, and normotensive groups, respectively. Although most preeclampsia studies report sEVs dose in micrograms rather than particles/cm², circulating sEVs concentrations in maternal plasma are commonly reported in the range of 10¹¹ particles/mL and are elevated in preeclampsia (Narumi et al., 2025). Thus, our experimental exposure lies within a biologically plausible range and provides a transparent quantitative context for interpreting endothelial responses.

### Cell viability assay

To analyze the effect of plasma on cell viability, we used the CellTiter 96 Non-Radioactive Kit (Lot: 0000105232, Promega, Madison, WI, USA) according to the manufacturer’s instructions. hCMEC/D3 cells (Merck Millipore, Darmstadt, Germany) were cultured on a 96-well plate and, after reaching 80% confluence, were treated with plasma (24 h, 1% v/v per well) from respective experimental groups. Absorbance was analyzed using an Epoch spectrophotometer (BioTek Instruments, VT, USA), set up at 570 nm (Leon et al., 2021).

### Cell proliferation

Cell proliferation was analyzed using a 5-bromo-2-deoxyuridine (BrdU, 10 mM) incorporation assay (Roche, INDY, USA) in hCMEC/D3 cells treated (1%, v/v, 24 h) with plasma from women with normal pregnancy and preeclampsia/eclampsia, as indicated above. The luminescence was quantified with an Epoch spectrophotometer (BioTek Instruments, VT, USA), with an absorbance of 540 nm.

### Plasma sEVs: isolation, characterization, and protein content

EDTA-plasma sEVs were isolated using a differential centrifugation and microfiltration protocol, as described previously (Thery et al., 2006; Leon et al., 2021; Sandoval et al., 2024). Sequential centrifugation of plasma diluted in 1X PBS (pH 7,4) was performed (Gardiner et al., 2016). Briefly, after collecting plasma, we performed sequential centrifugation steps: (1) 300 *x g* for 10 minutes, (2) 2000 *x g* for 10 minutes, (3) 10,000 *x g* for 30 minutes, and (4) 120,000 *x g* for 2 hours at 4 °C. The final supernatant was passed through a 0.22 μm filter and then centrifuged at room temperature at 120,000 *x g* for 18 hours. We recovered the pellet containing sEVs, resuspended it in PBS (pH 7.4), and passed it through a 0.22 μm filter. Finally, we performed the last centrifugation at 120,000 *x g* for 3 hours at 4 °C. The pellet was resuspended in PBS (pH 7.4, previously depleted of sEVs) and then again passed through a 0.22 μm filter.

Small extracellular vesicles were characterized by size and concentration using the NanoSight NS300 Instrument (Malvern Instruments, Ltd, Malvern, United Kingdom) (Mellisho et al., 2017). Samples were analyzed in a liquid suspension (PBS pH 7.4, 1:1000 dilution) at room temperature. Measurements ensured that there were between 20 and 100 particles per frame. Negative controls had fewer than seven particles per frame. Each sample was measured in triplicate with the same camera setup, and data were analyzed using NTA version 3.2 Dev Build 3.2.16 analytical software.

In addition, transmission electron microscopy (TEM) was performed on representative, randomly selected samples from women with normotensive pregnancies and preeclampsia, as previously described (Sandoval et al., 2024). Ten microliters of each sEVs sample (in 1X PBS) were placed on formvar-carbon-coated copper grids. Samples were fixed with 4% paraformaldehyde (1:1, 20 min), treated (6 min) with 1% glutaraldehyde, and washed with molecular-grade water. For contrast, grids were treated (5 min) with 0.5% uranyl oxalate (pH 7.0), dried at room temperature, and imaged at 40,000 X to 80,000 X magnification using a Jeol Jem 1200 EXII TEM with a Gatan 782 camera (5Å resolution, 80 kV).

Small extracellular vesicles were also characterized in terms of protein markers using Western blot and the following Santa Cruz Biotechnology antibodies (Dallas, TX, USA): Alix, sc-53540; CD63, sc-5275; CD81, sc-7637; TSG101, sc-7964; and HSP70, sc-66048. Also, placental alkaline phosphatase (PLAP, sc-53414) as a placental marker was analyzed (Leon et al., 2021; Sandoval et al., 2024).

### Protein content of BBB modulators in the plasma sEVs

Plasma sEVs protein extracts were used to analyze the presence of potential BBB modulators, including TNF-α (Santa Cruz Biotechnology, sc-52746) and eNOS (BD Transduction Laboratories, Becton, NJ, USA; 610299), by Western blot. For these Western blot analyses, sEVs samples were randomly pooled within each clinical group (up to 3 individual samples per pool) prior to protein loading. This procedure was implemented exclusively to ensure sufficient protein yield and did not affect the number of biological replicates included in the quantitative analyses.

For Western blot, total protein quantification was performed using the Pierce ™ BCA Protein Assay Kit (Thermo Fisher Scientific, Waltham, MA, USA) according to the manufacturer’s instructions. 50 μg of sEVs protein were separated in a 10% SDS-PAGE gel, transferred to a nitrocellulose membrane, and incubated with the respective primary antibodies. Ponceau red staining was used as a protein loading control.

### sEVs uptake by hCMEC/d3 cells

To characterize the uptake of sEVs by hCMEC/D3 cells, the former were labeled with PKH67 Green Fluorescent Cell Linker Mini Kit (MINI67-1KT, Sigma-Aldrich, St. Louis, MO, USA), according to the manufacturer’s protocol and cleared of free dye through an Amicon^®^ Ultra Centrifugal Filter unit -100 kDa MWCO (Merck; Darmstadt, Germany). Briefly, 100 µg/ml of labeled sEVs were applied to hCMEC/D3 monolayers for 1 hour at 37 °C. Cells were then fixed with 4% paraformaldehyde (PFA) in PBS 1x for 20 minutes at room temperature. Over several PBS washes, slides were incubated with DAPI for 20 minutes and mounted on microscopic slides using DAKO mounting medium. After this, slides were kept at 4 °C until analysis by fluorescent microscopy (40X magnification) (Motic Scientific, San Antonio, TX, USA). To quantify the uptake of sEVs by hCMEC/D3 cells, the presence of green dots (i.e., sEVs) at the FITC channel was measured.

In parallel experiments, to confirm sEVs on cell uptake, we used the latest generation of fluorescent labeling reagent to specifically label sEVs RNA cargo (ExoGlow™ kit, System Biosciences, Palo Alto, CA, USA) in hCMEC/D3 at 37°C (active incorporation) and 4 °C (nonactive incorporation, background) as previously described with PKH67 (Sandoval et al., 2024), and following manufacture instructions.

### Statistics

All statistical analyses were performed using Prism (GraphPad Software, La Jolla, CA, USA; version 10). The Shapiro-Wilk normality test was used to discriminate between parametric and non-parametric distributions. The unpaired t-test (parametric) or the Kruskal-Wallis test (non-parametric), followed by an uncorrected Dunn’s test, was used accordingly. An ordinary one-way ANOVA was used to evaluate differences in clinical variables. Clinical variables are expressed as mean ± S.D. Experimental results are reported as median ± interquartile range. A p-value of 0.05 was set as the threshold for statistical significance. All experiments were performed in duplicate.

## Results

### Background characteristics

Baseline characteristics of the study participants are presented in **Table 1**. Ordinary one-way ANOVA showed statistical differences in maternal age, body mass index, gestational age at birth, neonatal birthweight, and placental weight (all p<0.05). Notably, women with eclampsia were younger (p<0.05), with 65% being nulliparous, and delivered earlier (p<0.05) than normotensive controls. Mean newborn birthweight and placental weight were low in the preeclampsia and eclampsia groups compared to normal pregnancies (all p<0.05). Adverse perinatal outcomes, including intrauterine and neonatal deaths, occurred in 10 cases, predominantly among women with eclampsia and complicated preeclampsia.

**Table 1:** Characteristics of included women.

	Normotensive Controls n=18	Preeclampsia n=19	Preeclampsia with complications n=17	Eclampsia n=20
Baseline characteristics				
Maternal age, years ± SD	27.5 ± 7.0	26.9 ± 5.5	30.6 ± 5.8	23.7 ± 5.2‡
Nulliparous, n (%)	6 (33)	10 (53)	5 (29)	13 (65)
Chronic hypertension, n (%)	0 (0)	2 (10.5)	4 (25)	3 (15)
BMI (kg/m^2^), mean ± SD	25.2 ± 6.8	28.5 ± 7.4	32.1 ± 9.5*	25.8 ± 4.6
At inclusion				
Magnesium sulfate, n (%)	2 (11)	18 (95)	17 (100)	20 (100)
At birth				
Gestational age at birth (week + days)	35 + 2 (4 + 2)	32 + 1 (4 + 2)*	31 + 6 (3 + 6)*	31 + 2 (4 + 6)*
Mode of birth				
Vaginal n (%)	5 (28)	6 (32)	2 (12)	7 (35)
Planned caesarian, n (%)	11 (61)	3 (16)	1 (6)	0 (0)
Emergency caesarian, n (%)	2 (11)	10 (52)	14 (82)	13 (65)
Birthweight, gr, mean ± SD	2522.5 ± 997.9	1666.8 ± 975.0*	1636.7 ± 881.5*	1594 ± 845.0*
Sex of newborn female, n (%)	5 (28)	7 (37)	9 (53)	11 (55)
Placental weight, g, mean ± SD	555.7 ± 158.6	381.6 ± 174.4*	393.7 ± 159.3*	363.8 ± 177.2*
Complications				
Recurrent eclampsia, n (%)	N/A	N/A	N/A	6 (30)
Severe hypertension, n (%)	0 (0)	6 (32)	13 (77)	11 (55)
Stroke, n (%)	0 (0)	0 (0)	0 (0)	1 (5)
Cortical blindness, n (%)	0 (0)	0 (0)	2 (12)	1 (5)
Creatinine > 120, n (%)	0 (0)	0 (0)	4 (24)	3 (15)
Dialysis, n (%)	0 (0)	0 (0)	1(6)	0 (0)
Pulmonary edema, n (%)	0 (0)	0 (0)	13 (77)	1 (5)
Inotropic support, n (%)	0 (0)	0 (0)	0 (0)	0 (0)
HELLP, n (%)	0 (0)	0 (0)	4 (24)	6 (30)
Postpartum hemorrhage, n (%)	1 (6)	0 (0)	2 (12)	1 (10)
GCS <13	0 (0)	0 (0)	0 (0)	7 (35)
Intrauterine fetal death, n (%)	0 (0)	2 (11)	2 (12)	3 (15)
Neonatal death, n (%)	0 (0)	0 (0)	2 (12)	1 (5)
Placental abruption, n (%)	1 (6)	0 (0)	2 (12)	1 (5)

Glasgow coma scale (GCS). SD standard deviation. Data were analyzed using one-way ANOVA followed by Tukey’s *post hoc* test when appropriate. Analyses were performed using GraphPad Prism version 10. Symbols indicate statistical significance as follows: *p < 0.05 versus Normal Pregnancy; †p < 0.05 versus Preeclampsia; ‡p < 0.05 versus Preeclampsia with complications.

From this initial description of the included women, we must note that sample sizes varied across *in vitro* experiments (see below) due to the limited volume and exceptional clinical value of plasma obtained from women with preeclampsia or eclampsia. For each analysis, plasma or plasma-derived sEVs were randomly selected from the available cohort, and group sizes are reported in the corresponding figure legends. Reduced sample numbers in specific experiments reflect technical requirements, including minimum volume, sEVs yield, and quality control criteria (e.g., absence of hemolysis and sufficient sEVs concentration). No samples were excluded based on experimental outcomes.

### Effect of plasma on the BBB *in vitro* model

**Figure 1:**
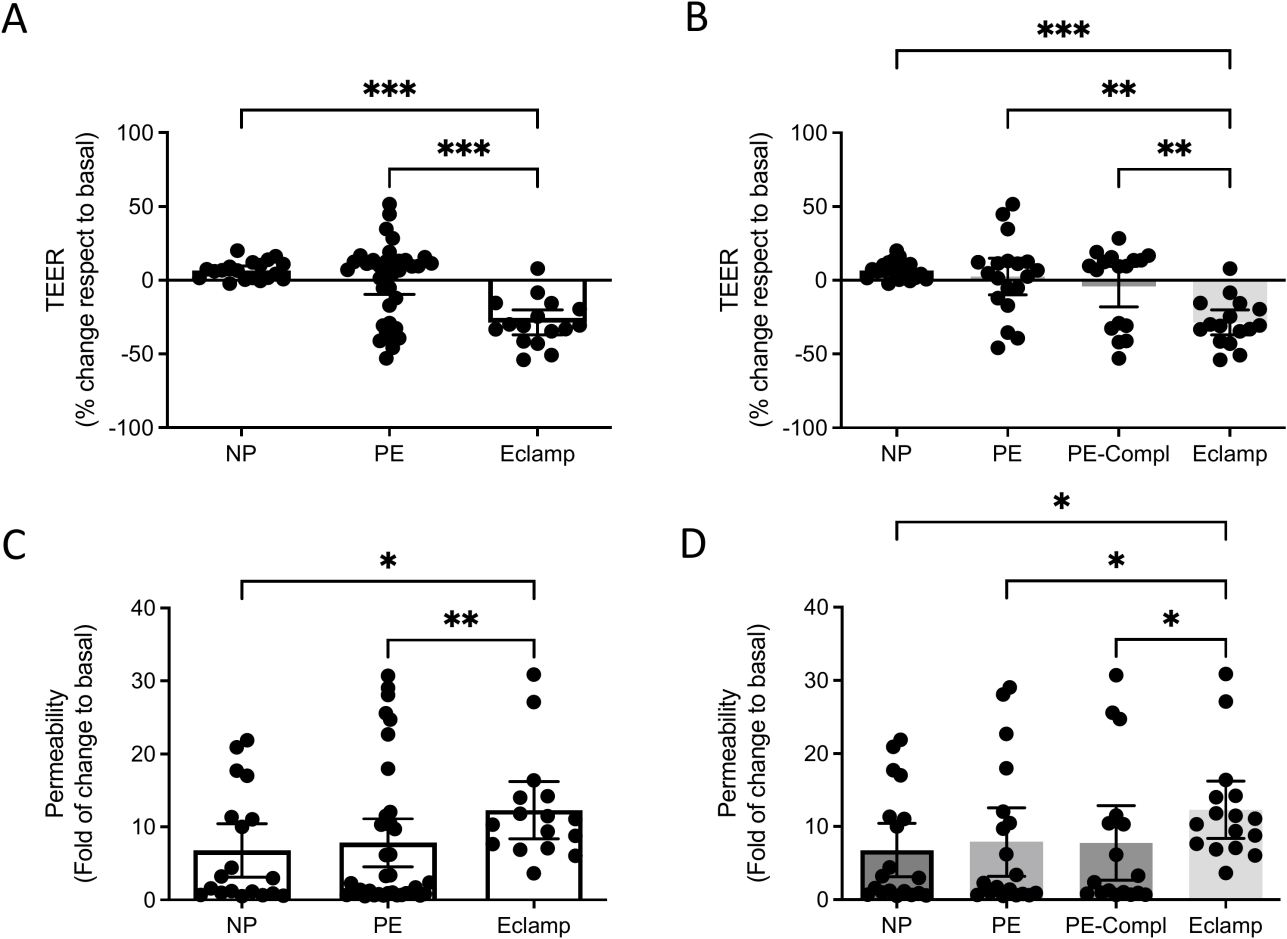
Effect of plasma of women with preeclampsia or eclampsia on the BBB *in vitro* model. **(A, B)** TEER and **(B, D)** Dextran 70 kDa permeability were analyzed in hCMEC/D3 cultures exposed (12 h, 1% v/v) to plasma of women with eclampsia (Eclamp, n=16), preeclampsia with (PE-Compl, n=17) or without (PE, n=19) organ complications, and normotensive controls (NP, n=19). Every dot represents an individual subject. Values in A and B are expressed as a percentage of change to basal (i.e., no treatment), whereas **(C, D)** are expressed as a fold of change to the basal condition. Values are reported as median ± interquartile range. *p<0.05. **p<0.005; ***P<0.0005. Kruskal-Wallis’ test, followed by Dunn’s multiple comparisons test.

We analyzed whether plasmas of women with preeclampsia or eclampsia can disrupt the BBB *in vitro* model. Our results indicate that plasma from women with eclampsia disrupted the BBB model compared with the plasma of women with preeclampsia or the plasma of women with normal pregnancy, as evidenced by a higher drop in the TEER values (**Figure 1A**, Kruskal-Wallis test, p=0.0003) and higher 70 kDa Dextran permeability assay (**Figure 1C**, Kruskal-Wallis test, p=0.049).

Then, we stratified the preeclampsia group into those with and without organ complications. TEER (**Figure 1B**) and permeability (**Figure 1D**) were significantly impaired in plasma from women with eclampsia compared to those with preeclampsia complicated by organ involvement. Significantly, plasma from women with preeclampsia, regardless of the presence or absence of complications, did not significantly alter BBB integrity markers compared to plasma from women with normal pregnancies.

**Figure 2:**
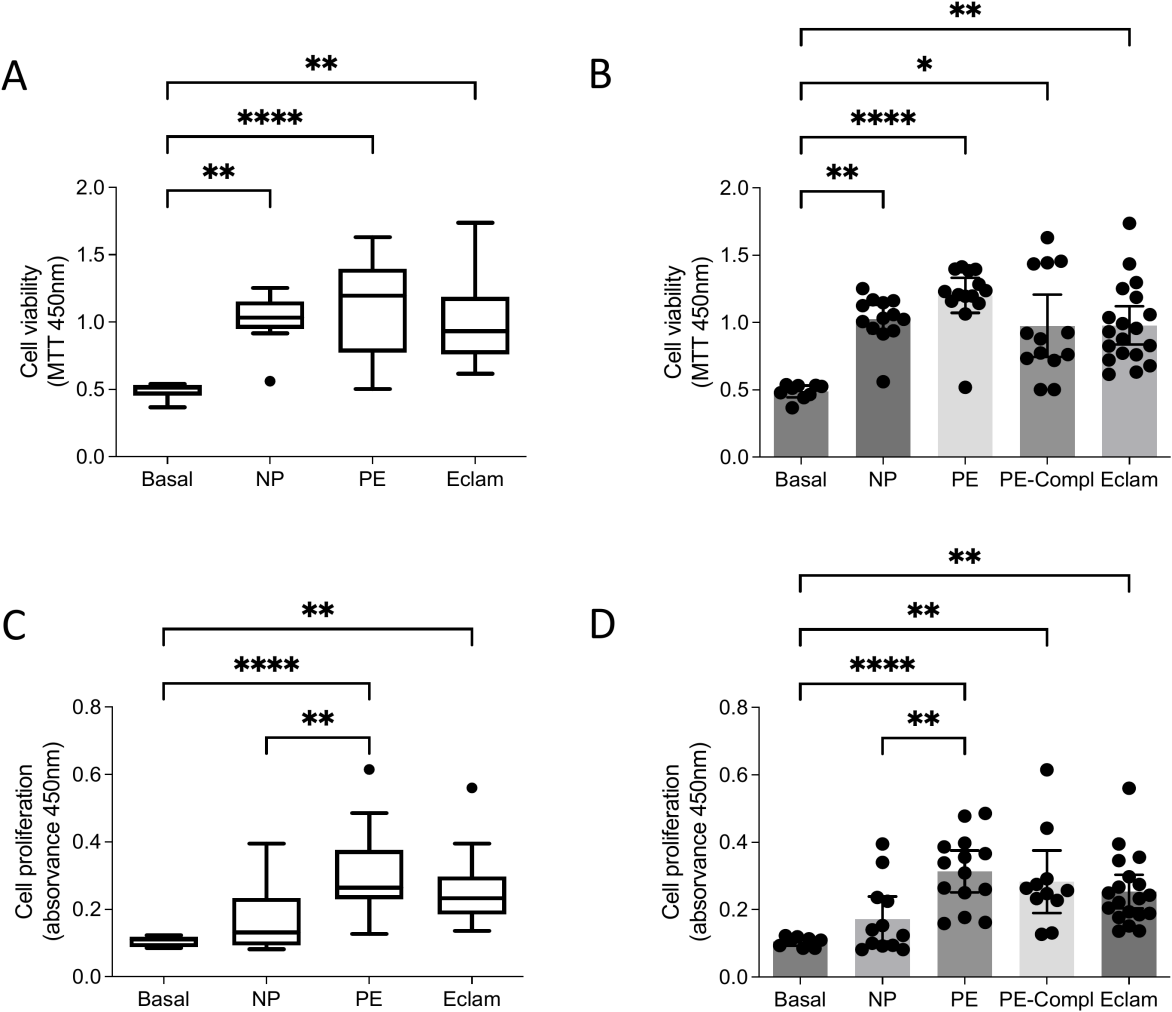
Effects of plasma of women with preeclampsia or eclampsia on hCMEC/D3 cells viability and proliferation. **(A, B)** Cell viability measured by MTT assay, and **(C, D)** Cell proliferation measured by BrU incorporation in brain endothelial cells (hCMEC/D3) exposed (24 h, 1% v/v) to plasma of women with eclampsia (Eclamp, n=19), preeclampsia with (PE-Compl, n=13) or without (PE, n=14) organ complications and normotensive controls (NP, n=13). Basal condition (i.e., no treatment). Every dot represents an individual subject. Values are expressed in absorvance units. Values are presented as medians with interquartile ranges. *p<0.05. **p<0.005; ****P<0.0001. Kruskal-Wallis ‘ test, followed by Dunn’s multiple comparisons test.

To determine whether the BBB disruption induced by plasma from women with eclampsia was related to reduced cell viability, we assessed mitochondrial activity (MTT assay) and cell proliferation (BrdU incorporation) in brain endothelial cells. Plasma from all study groups significantly increased mitochondrial activity, indicative of enhanced cell viability, compared with basal conditions (unstimulated cells; Kruskal–Wallis test, p<0.0001; **Figures 2A, B**). Similarly, compared with basal conditions, cell proliferation was significantly elevated in response to plasma from women with eclampsia and those with preeclampsia, regardless of systemic involvement (Kruskal–Wallis test, p<0.0001; **Figures 2C, D**). Notably, plasma from women with preeclampsia without systemic involvement induced greater proliferation than plasma from women with normal pregnancies. These results suggest that the BBB disruption induced by plasma from women with eclampsia is not driven by endothelial cell loss or reduced viability.

### Effect of the plasma-derived sEVs on the *in vitro* BBB model

**Figure 3:**
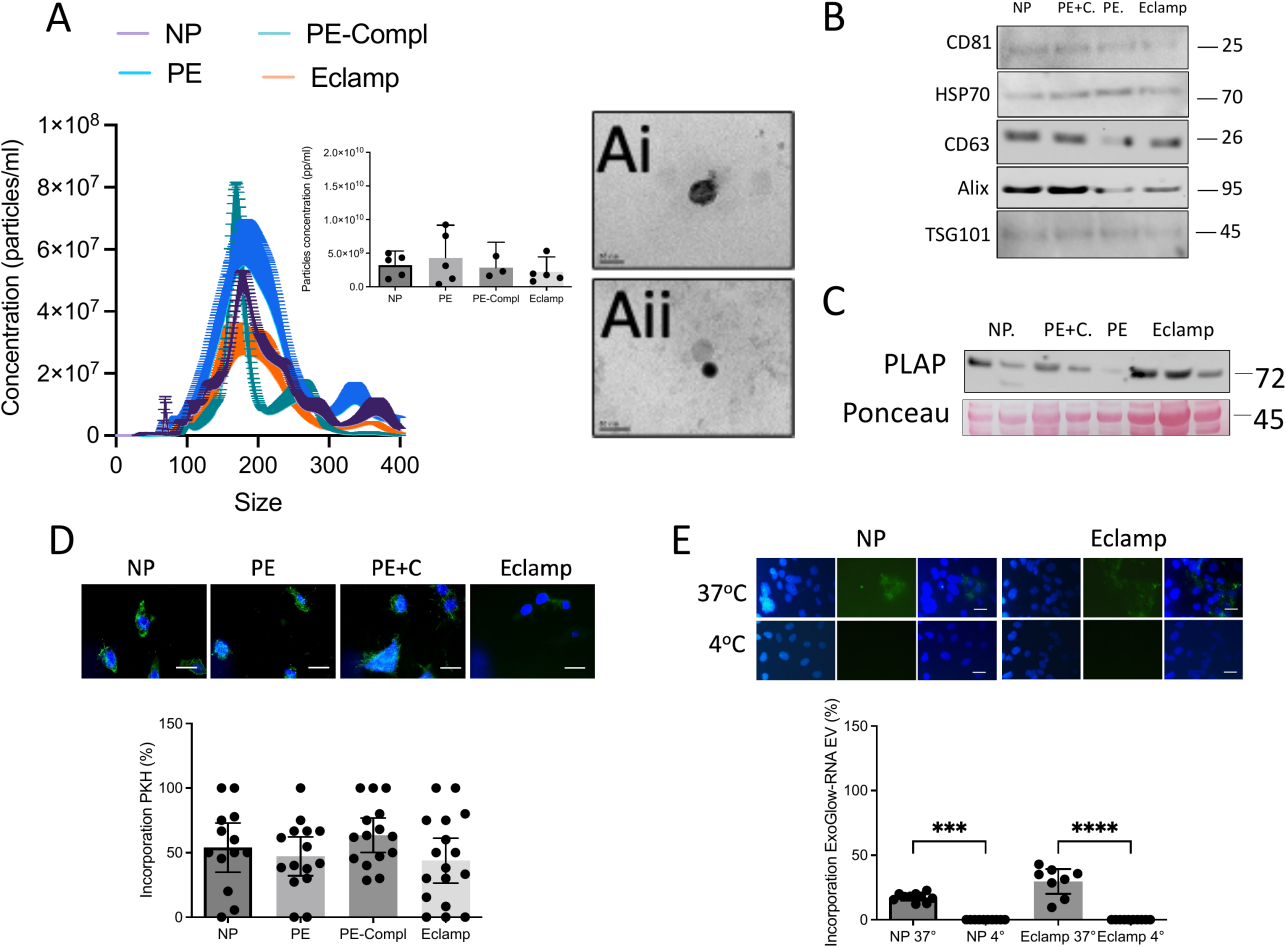
Characterization of sEVs isolated from plasma of women with preeclampsia or eclampsia. sEVs were isolated from plasma of women with eclampsia (Eclamp, n=5), preeclampsia with (PE-Compl, n=5) or without (PE, n=5) organ complications, and normotensive controls (NP, n=5) using the ultracentrifugation and filtration method (see Methods). **(A)** Chart showing the size and concentration distribution of sEVs from the experimental groups. The inserted graph indicates particle concentration. Images are representative sEVs observed in transmission electron microscopy (TEM) from randomly chosen normotensive pregnancy (Ai) or preeclampsia (Aii) groups. Line in the images, 50 nm. **(B)** Representative images of sEVs marker (HSP70, CD81, Alix, TSG101, CD63). **(C)** Representative image of placental marker (PLAP). The loading control was Ponceau Red staining. **(D)** sEVs (100 μg/ml) were treated with a fluorescent dye (PKH67) as indicated in Methods and used to estimate the percentage of cells that uptake sEVs (i.e., PKH67 positive) in each visual camp. **(E)** Representative sEVs incorporation using ExoGlow-RNA selective marker at 37 °C (active incorporation) and 4 °C (background). In the graph ***P<0.0005 and ****P<0.0001. Every dot represents an individual subject. Values are reported as median ± interquartile range.

To assess whether sEVs present in the plasma of women with eclampsia contribute to the BBB disruption observed with whole plasma, we first isolated sEVs from all studied groups. Nanoparticle tracking analysis showed no significant differences in mean particle size or total particle count among groups (**Figure 3A**). The isolated sEVs were further characterized by electron microscopy and Western blotting for exosomal markers (**Figure 3B**). No significant group differences were found in the relative levels of the placental marker PLAP (**Figure 3C**) or in cellular uptake efficiency (**Figure 3D**). Importantly, active incorporation of sEVs (37 °C) from both normal pregnancy and eclampsia plasma into hCMEC/D3 cells was confirmed by detecting specific sEVs RNA cargo in recipient cells (**Figure 3E**).

To evaluate whether MgSO_4_ may influence sEVs uptake, we quantified the percentage of hCMEC/D3 cells incorporating fluorescently labeled sEVs. MgSO_4_ pretreatment (−3 h) significantly reduced sEVs uptake in the normotensive pregnancy and preeclampsia groups (p = 0.0028), whereas no significant reduction was detected in the eclampsia group (**Supplementary Figure 1**).

**Figure 4:**
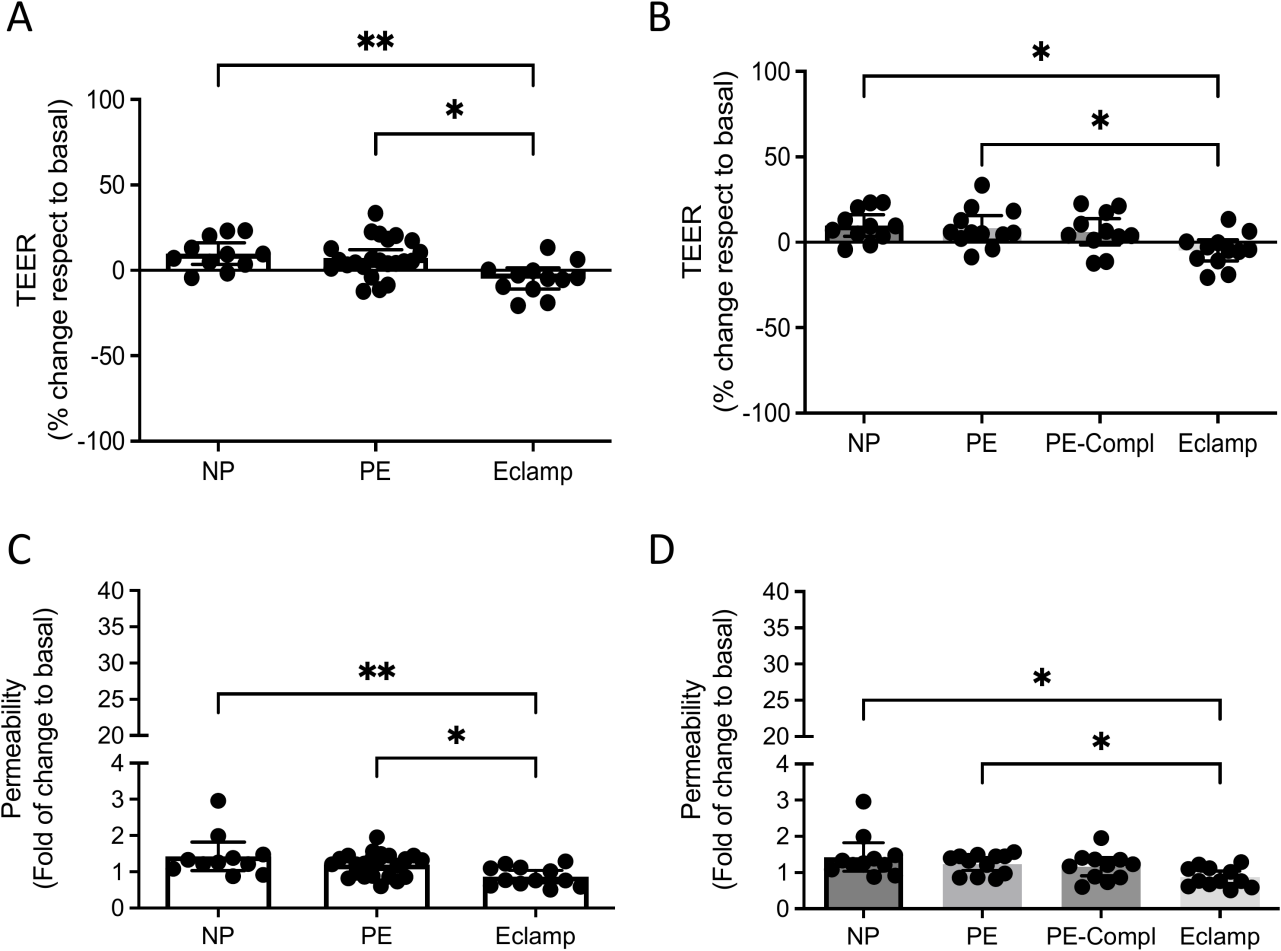
Effect of sEVs isolated from women with preeclampsia or eclampsia on the BBB *in vitro* model. sEVs isolated from the plasma of women with eclampsia (Eclamp, n=12), preeclampsia with (PE-Compl, n=11) or without (PE, n=12) organ complications, and normotensive controls (NP, n=11) were used. **(A, B)** TEER and **(B, C)** Dextran 70 kDa permeability in hCMEC/D3 cell monolayers after treatment (12 h, 100 μg). Every dot represents an individual subject. Values in A and B are expressed as a percentage of change to basal (i.e., no treatment), whereas **(C, D)** are expressed as a fold of change to the basal condition. Values are reported as median ± interquartile range. *p<0.05; **p<0.005. Kruskal-Wallis’ test, followed by Dunn’s multiple comparisons test.

Consistent with the effects observed for whole plasma (**Figure 1**), plasma-derived sEVs from women with eclampsia caused a significant reduction in TEER compared to sEVs from women with normal pregnancy or preeclampsia (Kruskal–Wallis test, p = 0.0095; **Figure 4A**). This reduction was not significantly different between sEVs from eclampsia and those from preeclampsia with organ complications (**Figure 4B**).

Conversely, eclampsia-derived sEVs showed lower permeability in hCMEC/D3 cells than those from normal pregnancy or preeclampsia (**Figure 4C**). Moreover, no significant differences in permeability were observed between eclampsia and preeclampsia with organ complications (**Figure 4D**).

Complementary analysis (**Supplementary Figure 2**) of automated quantification of F-actin length in endothelial cells exposed to sEVs derived from the plasma of women with preeclampsia without complications revealed a significant increase in fiber length compared with normal pregnancy. This increase was not observed in cells treated with sEVs from women with eclampsia or from preeclampsia with complications. No significant differences were detected between the eclampsia and normal pregnancy groups.

### Content of sEVs

**Figure 5:**
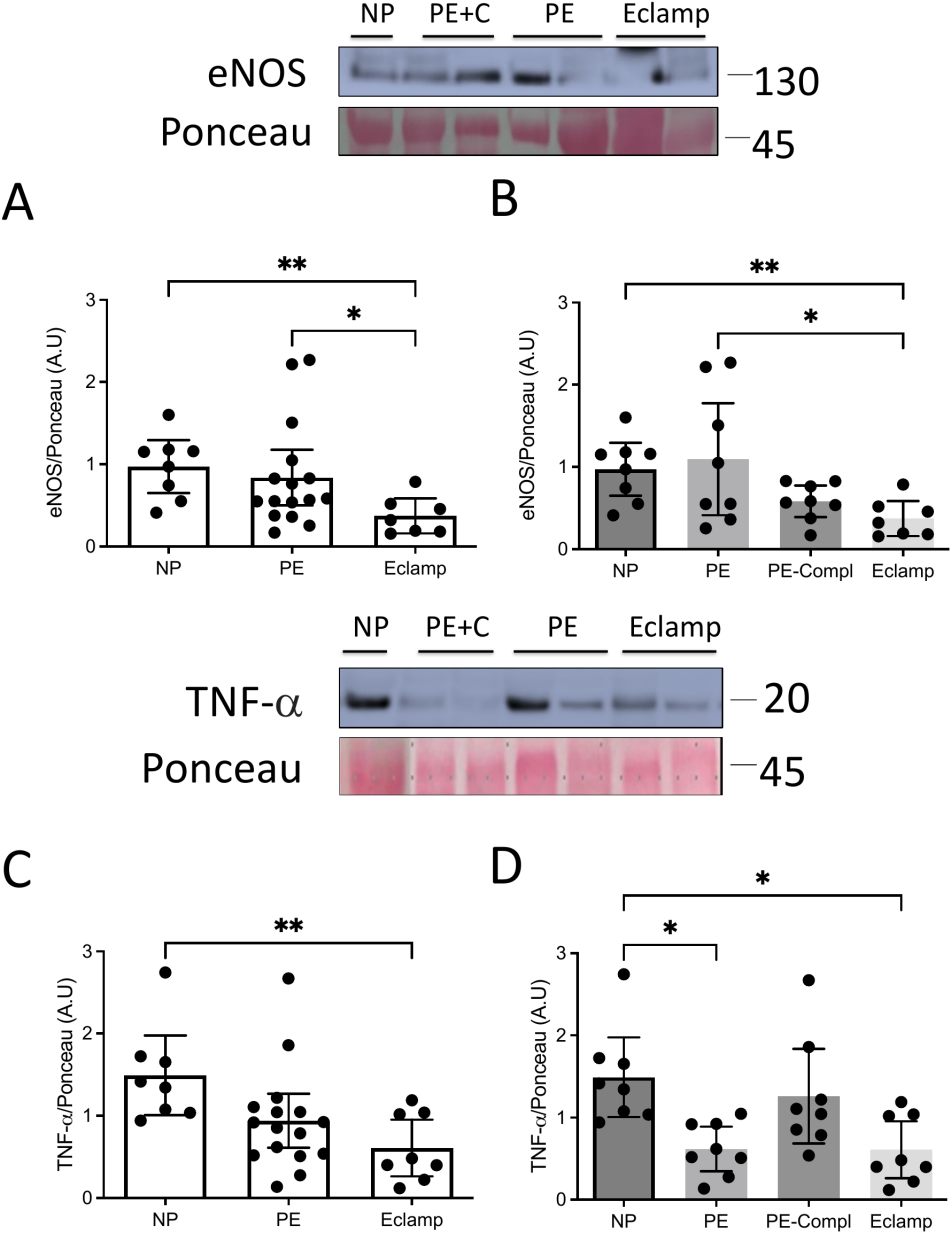
Analysis of the cargo of sEVs of BBB modulatory proteins. **(A, B)** Representative images of Western blot for eNOS, or **(C, D)** TNF-α present in sEVs from the plasma of women with eclampsia (Eclamp, n=8), preeclampsia with (PE-Compl, n=8) or without (PE, n=9) organ complications, and normotensive controls (NP, n=8). Ponceau staining was used to assess cargo loading. *P<0.05. **P<0.005. Kruskal-Wallis’ test followed by Uncorrected Dunn’s test.

To evaluate whether the unexpected decrease in permeability observed with eclampsia-derived sEVs was associated with its cargo, we analyzed protein levels of eNOS and TNF-α in sEVs. Western blot analysis of plasma-derived sEVs showed lower levels of eNOS (**Figure 5A**) and TNF-α (**Figure 5C**) in eclampsia compared to normotensive pregnancy or preeclampsia. No significant differences were found between eclampsia and preeclampsia with organ complications for either marker (**Figures 5B, D**).

## Discussion

Our findings demonstrate that plasma from women with eclampsia significantly disrupts BBB integrity, as evidenced by reduced TEER values and increased macromolecular permeability in brain endothelial cells. Notably, these effects appear independent of changes in cell viability or cell proliferation. In addition, plasma-derived sEVs from the same women with eclampsia induced less BBB impairment than those from preeclampsia or normotensive pregnancies, suggesting that soluble plasma factors, rather than sEVs, are the primary drivers of BBB dysfunction in eclampsia. This attenuated effect of sEVs may be due to decreased levels of eNOS and TNF-α within the sEVs cargo. A key strength of this work is the inclusion of plasma from women with eclampsia, a life-threatening pregnancy complication that remains a cause of maternal mortality even in high-income countries; access to such samples is exceptionally limited, and their analysis in a human BBB-focused framework provides rare insight into the cerebrovascular consequences of severe hypertensive disorders of pregnancy.

To our knowledge, no previous studies have directly examined whether plasma from women with eclampsia disrupts BBB integrity *in vitro*. Our data extend prior evidence from preeclampsia, where plasma has been shown to impair BBB function in both animal and human endothelial cell models (Amburgey et al., 2010; Schreurs et al., 2013; Bergman et al., 2021a; Leon et al., 2021; Friis et al., 2022; Torres-Vergara et al., 2022). These findings support the concept that circulating factors contribute to a pathophysiological cascade from BBB breakdown to neuronal hyperexcitability and eclamptic seizures (Johnson et al., 2018).

Currently, it is unclear which specific plasma component is responsible for BBB disruption *in vitro*. In rat cerebral veins, EDTA plasma (20%, v/v, 3 h) from women with preeclampsia increased BBB permeability, with a greater effect in early-onset (<34 weeks of gestation) than in late-onset (>34 weeks of gestation) disease. Notably, this effect was prevented by blocking LOX-1, the oxidized LDL receptor (Schreurs et al., 2013). Using a similar approach, this group reported that blocking VEGFR2 also prevents the increase in BBB permeability induced by plasma from women with preeclampsia (pooled plasma from women with early- and late-onset preeclampsia) (Amburgey et al., 2010). Building on those analyses, using human brain endothelial cells, we previously showed that heparin plasma from late-onset preeclampsia disrupted the *in vitro* BBB model, an effect also prevented by VEGFR2 inhibition (Bergman et al., 2021a; Torres-Vergara et al., 2022). Notably, in this model, TEER reduction correlated with higher circulating neurofilament light chain (NfL) levels, linking the *in vitro* findings to cerebral injury markers detected in women with preeclampsia (Friis et al., 2022). Collectively, these data suggest that LOX-1 and VEGFR2 activation are required for preeclampsia-associated BBB disruption. Although oxLDL and VEGF, the canonical ligands for these receptors, are altered in preeclampsia, the mechanistic interplay between them remains unclear. Additionally, these results did not exclude that other potential regulators, such as circulating TNF-α (Cipolla et al., 2012; Warrington et al., 2015), or AT1-AA (Duncan et al., 2020), which may also interplay in the generation of the BBB disruption observed in preeclampsia/eclampsia, as has been tested in animal models of this disease.

Notably, the absence of significant BBB disruption in cells exposed to plasma from South African women with preeclampsia with or without organ complications suggests that eclampsia involves distinct pathophysiological mechanisms beyond those observed in preeclampsia, at least in this population. This contrasts with our previous work showing that heparinized plasma from Swedish women with late-onset preeclampsia impaired BBB integrity in the same *in vitro* model (Bergman et al., 2021a). In the present study, the effect on the BBB induced by EDTA plasma from South African women with preeclampsia did not differ from that of women with normotensive pregnancies. Several anticoagulant-dependent factors may explain these divergent findings. First, heparin and EDTA differentially modulate plasma cytokine and chemokine profiles through distinct effects on leukocyte and platelet activation (Patil et al., 2013), potentially altering endothelial responses. Second, the choice of anticoagulant affects the availability of angiogenic and vascular mediators. For instance, heparin mobilizes soluble VEGF receptor-1 (sFlt-1) from heparan-sulfate complexes (Hagmann et al., 2014), shifting the VEGF/sFlt-1 balance toward barrier disruption, whereas EDTA does not (Park and Lee, 1999). Third, complement activation, which increases endothelial permeability, is preserved or enhanced under low-heparin conditions but is strongly inhibited by EDTA via Ca^2+^ chelation. Finally, anticoagulants differentially influence the sEVs populations and particularly their cargo recovered from plasma (Taha, 2023). These observations underscore the need to standardize or explicitly report anticoagulant type when assessing plasma-mediated BBB effects across cohorts.

Prior reports have shown that plasma-derived sEVs contribute to endothelial dysfunction and BBB impairment in preeclampsia (Cronqvist et al., 2017; Chang et al., 2018; Leon et al., 2021; Villalobos-Labra et al., 2022; Sandoval et al., 2024). Consistent with these observations, we found that sEVs from women with eclampsia induced a significant reduction in TEER compared with sEVs from normotensive pregnancies or preeclampsia, indicating altered endothelial electrical properties. TEER represents an indirect and highly sensitive electrical measurement of monolayer integrity that does not always correlate proportionally with macromolecular permeability. TEER values are influenced not only by tight junction continuity but also by junctional length, cellular morphology, and cytoskeletal organization (Felix et al., 2021). Previous studies using hCMEC/D3 cells have shown that sEVs-induced reductions in TEER may not be accompanied by sustained increases in permeability, particularly when barrier alterations are transient or temporally restricted (Roig-Carles et al., 2021). Although time-dependent analyses would further clarify this relationship, experimental conditions were standardized according to previously published work (Leon et al., 2021; Sandoval et al., 2024). Also, TEER reflects ion channel–dependent regulation of endothelial membrane resistance. Therefore, our findings suggest that eclampsia-derived sEVs may disrupt BBB function through mechanisms involving impaired monolayer cohesion and/or dysregulation of endothelial ion channels. In support of this interpretation, circulating factors in severe hypertensive disorders of pregnancy, including HELLP (Hemolysis, elevated liver enzymes, and low platelet count) syndrome, have been shown to blunt small and intermediate potassium (SK/IK) channel–mediated vasodilation in cerebral arteries, consistent with defective endothelial-derived hyperpolarization (Wallace et al., 2015). Together, these data support a model in which plasma components (including but not limited to sEVs) interfere with endothelial ion channel signaling, leading to electrical instability and BBB disruption, as captured by the reduction in TEER observed in our *in vitro* model.

Nevertheless, this sEVs-mediated reduction in TEERs was not correlated with increased permeability in cells exposed to sEVs from women with eclampsia, which raises a controversy with our previous findings using sEVs from the plasma of women with preeclampsia (Leon et al., 2021; Sandoval et al., 2024). In general, our results in the current manuscript, using plasma from women with eclampsia, indicate that the effect of sEVs was weaker than that of plasma on these two markers of the BBB *in vitro* across all experimental groups. Several factors may explain this attenuated effect, including technical variables such as anticoagulant type (heparin vs. EDTA), prior MgSO_4_ treatment (see below) (Leon et al., 2021), gestational age at sEVs isolation (Salomon et al., 2014), and pre-analytical handling (e.g., freeze–thaw cycles) known to influence sEVs cargo and function (Gelibter et al., 2022). In the present study, plasma samples were collected in EDTA tubes and processed identically across groups, minimizing systematic bias. At the 1% (v/v) concentration used in Transwell experiments, the estimated final EDTA concentration (~0.00015% w/v) is substantially below levels reported to disrupt barrier integrity, which typically require higher concentrations or combination with additional permeation-enhancing agents (Tomita et al., 1996; Greimel et al., 2007; Hu et al., 2016; Wang et al., 2012). Consistent with our previous studies using an alternative anticoagulant (i.e., heparin), these findings suggest that EDTA at this concentration is unlikely to explain the disease-specific effects observed, although its use remains a methodological limitation. Nevertheless, it is possible that EDTA isolates a different population of sEVs compared to lithium heparin plasma (Taha, 2023).

Consistent with our observation, O’Brien et al. reported that extracellular vesicles of unspecified size derived from placental explants did not significantly impair endothelial pro-angiogenic function, implicating soluble plasma factors, particularly angiogenesis-modulating proteins, as the primary drivers of vascular dysfunction in preeclampsia (O’brien et al., 2017). Collectively, these data underscore the influence of biological, population, and disease-dependent variability in circulating sEVs composition (Taha, 2023), while supporting the concept that non-vesicular circulating components may play a central role in BBB impairment in both preeclampsia and eclampsia.

Quantitative analysis of F-actin organization revealed increased fiber length in endothelial cells exposed to sEVs from women with preeclampsia without complications compared to normal pregnancy and eclampsia. In contrast, no such increase was observed with sEVs derived from eclampsia. F-actin fiber elongation is typically associated with enhanced actomyosin tension and reinforcement of junctional anchoring (Felix et al., 2021), suggesting that in non-complicated preeclampsia, endothelial cells may mount a compensatory cytoskeletal response aimed at preserving barrier integrity.

In contrast, eclampsia-derived sEVs reduced TEER without inducing F-actin fiber elongation, indicating that barrier disruption occurs through mechanisms not reflected by actin remodeling in this study. This dissociation suggests that the decrease in electrical resistance is unlikely to be driven by cytoskeletal reinforcement and instead may involve alternative processes, such as junctional instability, which were not directly evaluated. Therefore, the mechanism underlying TEER reduction in response to eclamptic sEVs remains unresolved.

Notably, functional barrier alterations did not parallel absolute particle number, suggesting that qualitative differences in vesicle cargo or bioactivity, rather than vesicle abundance alone, determine endothelial responses. In this regard, sEVs from women with eclampsia exhibited a distinct cargo profile compared to those from preeclampsia or normotensive pregnancies. They contained lower levels of eNOS and TNF-α, both key mediators of endothelial regulation and BBB stability (Cipolla et al., 2012; Warrington et al., 2015; An et al., 2021), which may underlie their attenuated capacity to disrupt the BBB. Moreover, the observed reduction in TNF-α within eclampsia-derived sEVs may appear unexpected given the disease’s inflammatory environment (Teran et al., 2001; Aggarwal et al., 2019). However, extracellular vesicle cargo does not necessarily reflect circulating plasma levels and may instead represent selective packaging or altered vesicle biogenesis under severe pathological conditions. While this targeted approach does not provide a global proteomic profile, it allows mechanistic exploration of pathways with strong biological relevance.

All women with eclampsia received MgSO_4_ therapy, and blood samples were collected predominantly during infusion, raising the possibility that magnesium exposure could influence the observed endothelial responses (Rochelson et al., 2007). In our *in vitro* experiments, MgSO_4_ pretreatment reduced sEVs uptake in normotensive and preeclampsia groups but had no significant effect in eclampsia. These findings are consistent with our previous observations in an independent cohort of women with preeclampsia (Leon et al., 2021) and support the notion that magnesium exposure may attenuate sEVs–endothelial interactions rather than enhance them. However, because all eclamptic patients were treated, we cannot exclude the possibility that sEVs effects on BBB function might differ in the absence of MgSO_4_. Plasma magnesium levels were not measured, which represents a limitation. Furthermore, additional factors may contribute to variability in the observed responses, including differences in gestational age between groups, the use of EDTA as an anticoagulant, and disease-related variations in plasma composition.

This study has limitations. Sample sizes varied across experiments due to the rarity of eclampsia and preeclampsia with severe complications, and the finite volume of ethically obtained human plasma, necessitating strategic allocation across assays. However, sample selection was random and independent of biological response, and the consistency of BBB-disruptive effects induced by plasma of women with eclampsia across independent readouts supports the robustness of our findings. Mechanistically, although we demonstrate marked BBB dysfunction driven by circulating factors, the specific molecular pathways involved—including discrete plasma components, sEVs subpopulations, endothelial ion channel regulation, MgSO_4_ effect, and tight junction remodeling—remain to be defined. Paired analyses using plasma and sEVs from the same individuals with different anticoagulants would further clarify anticoagulant-dependent effects. In addition, although functional blocking experiments were beyond the scope of this study, prior work from our group demonstrated that VEGFR2 antagonism attenuates BBB disruption induced by plasma from women with preeclampsia (Torres-Vergara et al., 2022), whereas VEGFR2 inhibition does not reverse barrier dysfunction triggered by hypoxia-derived placental sEVs (Sandoval et al., 2024). Furthermore, TNF-α (Cipolla et al., 2012; Warrington et al., 2015), VEGFR2 signaling (Amburgey et al., 2010; Warrington et al., 2015; Bergman et al., 2021a), and AT1-AA (Duncan et al., 2020) have been implicated in plasma-mediated BBB disruption in preclinical models of preeclampsia. Together, these findings provide indirect mechanistic support for the contribution of circulating factors. At the same time, the present study was specifically designed to compare the relative effects of whole plasma and isolated sEVs fractions.

In conclusion, our study underscores the critical role of circulating plasma factors in the disruption of the BBB during eclampsia. Although plasma-derived sEVs have been linked to endothelial dysfunction, our findings suggest they are not the main drivers of the BBB impairment observed in eclampsia, likely because of altered cargo composition. These results offer new insights into the mechanisms of cerebrovascular complications in eclampsia and highlight the need to identify and characterize these plasma factors as potential biomarkers.

## Data Availability

The data that support the findings of this study are available from the corresponding author, Dr. Carlos Escudero, upon reasonable request.
