# Supplementary material for: In vitro evidence that plasma of women with eclampsia disrupts the blood-brain barrier
Source: Front Physiol. 2026 Apr 27;17:1778955. doi: 10.3389/fphys.2026.1778955 (PMC13158122; doi:10.3389/fphys.2026.1778955)
Supplement: Supplementary file 1 [file SupplementaryFile1.docx]

**Supplementary information**

***In vitro* evidence that plasma of women with eclampsia disrupts the blood-brain barrier.**

Jesenia Acurio^1†^, Felipe Troncoso^1†^, Estefanny Escudero-Guevara^1,2^, Hermes Sandoval^1,2^, Belen Ibañez^1,3^, Manu Vatish^4^, Pablo Torres-Vergara^5,6^, Lina Bergman^7,8,9,10^, Carlos Escudero^1,5,11^*.

*^1^ Vascular Physiology Laboratory, Department of Basic Sciences, Universidad del Bío-Bío, Chillán, Chile.*

*^2^ Doctorade Program in Biomedical Sciences, Universidad de Talca, Talca, Chile*

*^3^ Doctorade Program in Veterinarian Sciences, Universidad de Concepcion, Chillan, Chile*

*^4^ Nuffield Department of Women’s & Reproductive Health. University of Oxford, Women’s Centre, John Radcliffe Hospital, Oxford OX3 9DU, United Kingdom*

*^5^ Group of Research and Innovation in Vascular Health (GRIVAS Health), Chillan, Chile.*

*^6^ Departamento de Farmacia, Facultad de Farmacia, Universidad de Concepción, Concepción, Chile.*

*^7^ Department of Women's and Children's Health, Uppsala University, Uppsala, Sweden*

*^8^ Department of Obstetrics and Gynecology, Stellenbosch University, Cape Town, South Africa*

*^9^ Department of Obstetrics and Gynecology, Gothenburg University, Gothenburg, Sweden*

*^10^ Region Västra Götaland, Sahlgrenska University Hospital, Department of obstetrics and gynecology, Gothenburg, Sweden*

*^11^ Consortium for Research and Innovation Neurovascular (NEUROVAS), Chillan, Chile*

^†^ These two authors contribute equally to this manuscript

*Correspondence: Carlos Escudero, MD PhD

Vascular Physiology Laboratory

Group of Research and Innovation in Vascular Health

Basic Sciences Department

Faculty of Sciences

Universidad del Bio-Bio

Chillán, Chile

Phone: 56-42-2463256 / Mobile: 56-9-65655127

[cescudero@ubiobio.cl](mailto:cescudero@ubiobio.cl)

**Manuscript word count:** 9295

**Abstract word count:** 229

**Financial disclosure:** Fondecyt 1240295 and GI2301146 (Chile). LB is funded by Research grants from the Swedish Research Council, STINT, Märta Lundqvist Stiftelse, Swedish Society of Medicine, SSMF, Jane and Dan Olsson Stiftelse, and Wallenberg Center for Molecular and Translational Medicine.

**Conflict of interest:** none

**Number of figures:** 5

**Number of Tables:** Table 1

Supplementary information: Yes. Supplementary methods, and Figures (S1-S2).

**Number of references:** 59

**Keycode:** Blood-brain barrier; extracellular vesicles; eclampsia.

**Abbreviations:** AT1-AA, Angiotensin II type 1 receptor agonistic autoantibodies. Body mass index (BMI). BBB, Blood-brain barrier. eNOS, endothelial nitric oxide synthase. FITC, Fluorescein-5-isothiocyanate. Human brain endothelial cell line, hCMEC/D3. LOX-1, oxidized LDL receptor. NfL, neurofilament light chain. sEVs, small extracellular vesicles, or exosomes. TEER, Transendothelial electrical resistance. TNF-α, Tumor necrosis factor-α. VEGF, Vascular endothelial growth factor. VEGFR2, vascular endothelial growth factor receptor 2. sFlt-1, Soluble vascular endothelial growth factor receptor 1.

**Supplementary Methods**

**sEVs uptake by hCMEC/d3 cells and the effect of magnesium sulfate**

A subset of plasma-sEVs (n=3 per group) were randomly chosen to characterize the uptake of sEVs by hCMEC/d3 cells, the former were labelled with PKH67 Green Fluorescent Cell Linker Mini Kit (MINI67-1KT, Sigma-Aldrich, St. Louis, MO, USA), according to the manufacturer protocol and cleared of free dye through an Amicon® Ultra Centrifugal Filter unit -100 kDa MWCO (Merck; Darmstadt, Germany). Briefly, 100 µg/ml of labelled sEVs were applied to hCMEC/D3 monolayers for 1 hour at 37°C. Cells were then fixed with 4% paraformaldehyde (PFA) in PBS 1x for 20 minutes at room temperature. Over several PBS washes, slides were incubated with DAPI for 20 minutes and mounted on microscopic slides using DAKO mounting medium. After this, slides were kept at 4°C until analysis by fluorescent microscopy (40X magnification) (Motic Scientific, San Antonio, TX, USA). To quantify the uptake of sEVs by hCMEC/D3 cells, the presence of green dots (i.e., sEVs) at the FITC channel was measured.

In parallel experiments, to visualize the effect of sEVs on cell uptake, we pretreated cells (-3 hours) with magnesium sulfate (80 mg/L w/v) before adding sEVs, as previously reported (Leon et al., 2021). As internal controls, similar experiments were performed at 37 °C (active transport) and 4 °C (unspecific background) (Sandoval et al., 2024).

**F-actin disorganization**

A subset of plasma-sEVs (n=5 per group) was randomly chosen to analyze F-Actin fibers using phalloidin iFluor 488 (1:500 v/v dilution) (Abcam, Cambridge, UK; ab176753) in hCMEC/D3 cells cultured over a glass put in 24-well plates and treated as described above using plasma or plasma-sEVs for 6 hours. After overnight incubation at 4°C, samples were sequentially washed with 1X phosphate-buffered saline (PBS). Then, nuclear labeling (DAPI) was added at a 1:10,000 (v/v) dilution for 20 minutes. Finally, all samples (i.e., cultured on glass) were transferred onto a 26 x 76 mm slide (Knittel Glass, BS, DE) and covered with a glass coverslip supported by mounting medium for subsequent visualization by fluorescent microscopy (Motic model BA410, Motic, HK, China). Images were captured at 100X magnification with a resolution of 2580 x 1944 pixels per image. Three RGB chromatic channels (Red, Green, and Blue) were used, with their corresponding filters (FITC) (Ex 330-380), (TRITC) (Ex 450-490), and (DAPI) (Ex 510-560). Then, captured fluorescent cell images were analyzed in ImageJ (National Institutes of Health, NIH) using the Mexican Hat filter in each analysis, which preserves high frequencies and thus highlights the fibers. The Ride Detection plugins were applied to analyze the number and length of fibers. The length of the fibers was normalized by the area (i.e., pixels) of the respective cell.

**Supplementary figures**

**Figure S1. Effect of magnesium sulfate on sEV uptake by brain endothelial cells.**

**A)** Representative images of hCMEC/D3 cells incubated with PKH67-labeled sEVs (100 μg, 1 h, 37^o^C) isolated from eclampsia (ECL, n=3), preeclampsia with complications (PE-Compl, n=3), preeclampsia without complications (PE, n=3), and normotensive pregnancies (NP, n=3), with (+) or without (-) MgSO₄ pretreatment (80 mg/L w/v). **B)** Percentage of PKH67-positive cells per field. Each dot represents an individual quantification per field from three independent experiments. Data are expressed as a percentage of cells incorporating PKH67 per field. Data are presented as medians with interquartile ranges. *p < 0.05 (Kruskal–Wallis test followed by Fisher´s LSD test).

**Figure S2. Effect of plasma-derived sEVs from women with preeclampsia or eclampsia on endothelial F-actin organization.** Small extracellular vesicles (sEVs) isolated from plasma of women with eclampsia (Eclamp, n = 5), preeclampsia with organ complications (PE-Compl, n = 5), preeclampsia without complications (PE, n = 5), and normotensive pregnancies (NP, n = 5) were applied to hCMEC/D3 monolayers (12 h, 100 μg). F-actin fiber length per cell was quantified as an index of cytoskeletal organization. **A)** Representative phalloidin staining of hCMEC/D3 cells. **B–C)** Quantitative analysis of F-actin fiber length following treatment. Each dot represents an individual cell from five independent experiments. Data are expressed as fold change relative to the basal (untreated) condition and presented as median with interquartile range. *p < 0.05; Kruskal–Wallis test followed by Dunn’s multiple comparisons test.

**Cited references**

Leon, J., Acurio, J., Bergman, L., Lopez, J., Karin Wikstrom, A., Torres-Vergara, P., Troncoso, F., Castro, F.O., Vatish, M., and Escudero, C. (2021). Disruption of the Blood-Brain Barrier by Extracellular Vesicles From Preeclampsia Plasma and Hypoxic Placentae: Attenuation by Magnesium Sulfate. *Hypertension* 78**,** 1423-1433.

Sandoval, H., Ibañez, B., Contreras, M., Troncoso, F., Castro, F.O., Caamaño, D., Mendez, L., Escudero-Guevara, E., Nualart, F., Mistry, H.D., Kurlak, L.O., Vatish, M., Acurio, J., and Escudero, C. (2024). Extracellular vesicles from preeclampsia disrupt the blood-brain barrier by reducing claudin-5. . *Arteriosclerosis, Thrombosis, and Vascular Biology* 45**,** 298-311.
